# Primary Bone Lymphoma of the Spine: A Case Report Highlighting Diagnostic Complexities and Treatment

**DOI:** 10.7759/cureus.52524

**Published:** 2024-01-18

**Authors:** Eisuke Tsukamoto, Yoshitaka Nagashima, Yusuke Nishimura, Kyoko Kuwabara, Ryuta Saito

**Affiliations:** 1 Neurosurgery, Nagoya University Graduate School of Medicine, Nagoya, JPN; 2 Pathology, Komaki City Hospital, Komaki, JPN

**Keywords:** posterior spinal decompression, pathological vertebral fracture, posterior fixtation, diffuse large b cell lymphoma (dlbcl), primary bone lymphoma

## Abstract

The diagnosis of primary bone lymphoma (PBL) of the spine is challenging due to its nonspecific symptoms and radiographic features. This report details the case of an 81-year-old female who presented with lower limb weakness and thoracic pain, consequent to a vertebral pathological fracture and spinal cord compression. The initial surgical intervention revealed granulomas with caseous necrosis; however, a definitive diagnosis remained elusive. Following a third surgical procedure and further histopathological examination, the patient was finally diagnosed with diffuse large B-cell lymphoma. The therapeutic course following diagnosis involved chemotherapy, resulting in a marked improvement of the symptoms.

Previous studies have highlighted the diagnostic difficulties associated with PBL, reporting the frequent need for multiple biopsies to confirm the diagnosis due to the prevalence of necrosis, crush artifacts, or inadequate sample volume. While PBL of the spine has shown responsiveness to chemotherapy and radiation therapy, early surgical intervention is advocated in cases of severe spinal cord compression or vertebral instability. The presented case highlights the importance of making a definitive pathology diagnosis in cases of suspected PBL of the spine.

## Introduction

Primary bone lymphoma (PBL) of the spine is a rare clinical entity among primary non-Hodgkin's lymphomas. Diagnosis of primary PBL is often challenging due to the difficulty in pathological diagnosis and the non-specific nature of its symptoms and radiological features. Generally, PBL was characterized as sheets of large lymphocytes with irregular nuclear contours and variably prominent nucleoli; however, there have been some cases reported in the past in which only necrotic tissue was found on pathological examination [1,2]. Multiple biopsies were necessitated in 30% of cases due to issues such as necrosis, crush artifacts, or inadequate sample volume [1].

In the context of treating PBL, recent findings suggest that chemotherapy can serve as an efficacious therapeutic modality, contingent upon accurate diagnosis [3]. However, surgical intervention in the spine can provide more than just diagnostic confirmation of diagnosis. It plays an important role for urgent neurological symptoms [2]. Therefore, in the surgery for PBL of the spine, it is crucial to obtain an adequate tumor sample and, in addition, to achieve preserving neurological function in those cases presenting with neurologic symptoms by decompression and spinal stabilization.

In this article, we present a case of thoracic spine PBL that was challenging to diagnose and required three surgeries. This case demonstrates the diagnostic complexity of pathologic diagnosis of PBL caused by pathologic necrosis. Notably, this case is characterized by the strategic use of the intraoperative navigation system and intraoperative ultrasound. These technological assistants led to a successful clinical outcome.

## Case presentation

**Figure 1:**
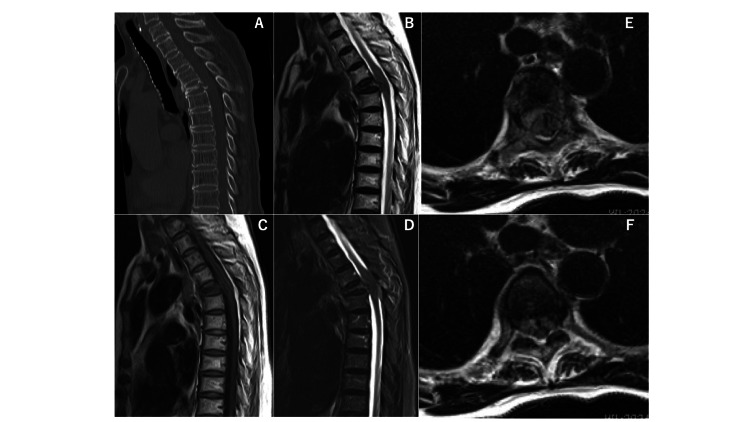
Computed tomography image and magnetic resonance images on arrival Computed tomography image (CT) shows a Th5 level vertebral fracture (A). Magnetic resonance images show the Th5 vertebral fracture and spinal compression at the same level (B: T2-weighted image, C: T1-weighted image, D: STIR. MRI T2-weighted axial images show spinal compression from the ventral side continuous vertebral body (E, F).

**Figure 2:**
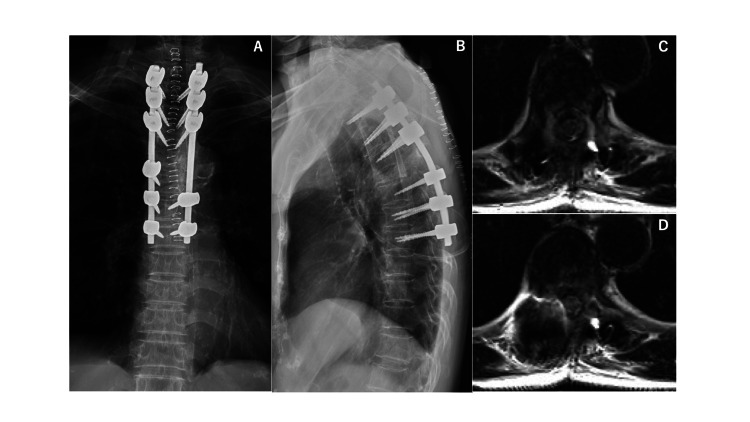
X-ray photographs and magnetic resonance images after the first surgery Frontal view (A) and lateral view (B) of X-ray images show good posterolateral fixation by screws and rods. After surgery, spinal compression by tumor is improved at the Th5 level (C, D).

**Figure 3:**
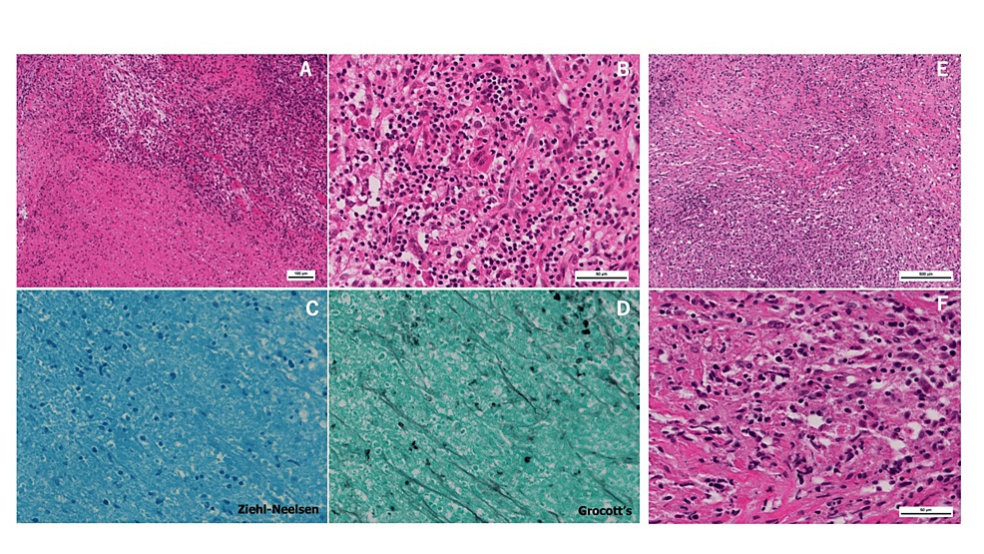
First and second surgical pathological specimens The first surgical specimen stained with hematoxylin-eosin showed necrosis and small lymphocytes (A: x100, B: x400). Tuberculosis examinations did not show positive staining for Ziehl-Neelsen (C) and Grocott’s (D). The second surgical specimen stained with hematoxylin-eosin had similar findings (E: x100, F: x400).

An 81-year-old female patient with a medical history of bladder cancer and tuberculosis, previously treated with total cystectomy, presented to our hospital due to weakness in both lower extremities and chest pain. Neurological examination revealed a manual muscle testing level of 2/5 in both lower extremities. Spinal computed tomography (CT) showed a vertebral fracture at the Th5 level. Spinal magnetic resonance imaging (MRI) revealed spinal cord compression due to pathological fracture at Th5 (Figure 1). An urgent surgical intervention was conducted, consisting of posterior decompression, posterior fixation from Th2 to Th8, and partial removal with tumor tissue sampling for analysis (Figure 2). These procedures were performed using CT-based navigation. Pathology results showed granuloma with caseous necrosis (Figure 3), and various tests (PCR, bacterial culture test, TSOPT, Gaffky) were negative for tuberculosis. Postoperatively, paralysis and pain showed improvement.

**Figure 4:**
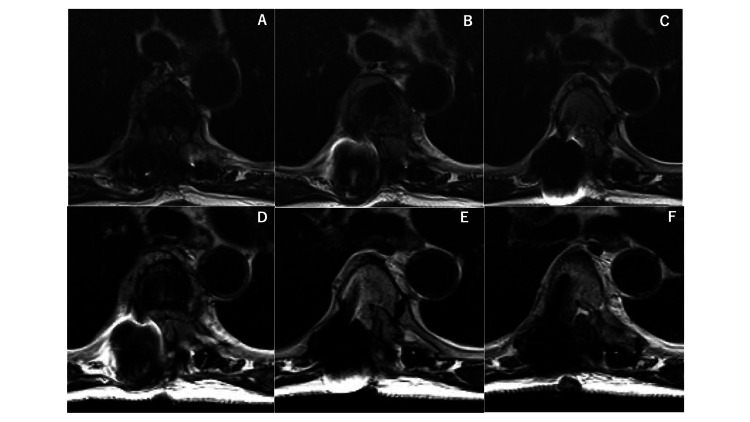
Magnetic resonance images before and after the second surgery MRI T2-weighted images show the recompression of the spinal cord by the tumor (A), leading to a second operation (B).

Approximately one and a half months postoperatively, the patient experienced a recurrence of symptoms in both lower limbs and chest. Repeat spinal MRI showed regrowth of the tumor and compression of the spinal cord. A second tumor resection was thus performed (Figure 4), which temporarily ameliorated her symptoms. However, the second surgical specimen also showed that the lymphocytes were relatively small and did not show atypia, so a definitive diagnosis remained elusive (Figure 3).

**Figure 5:**
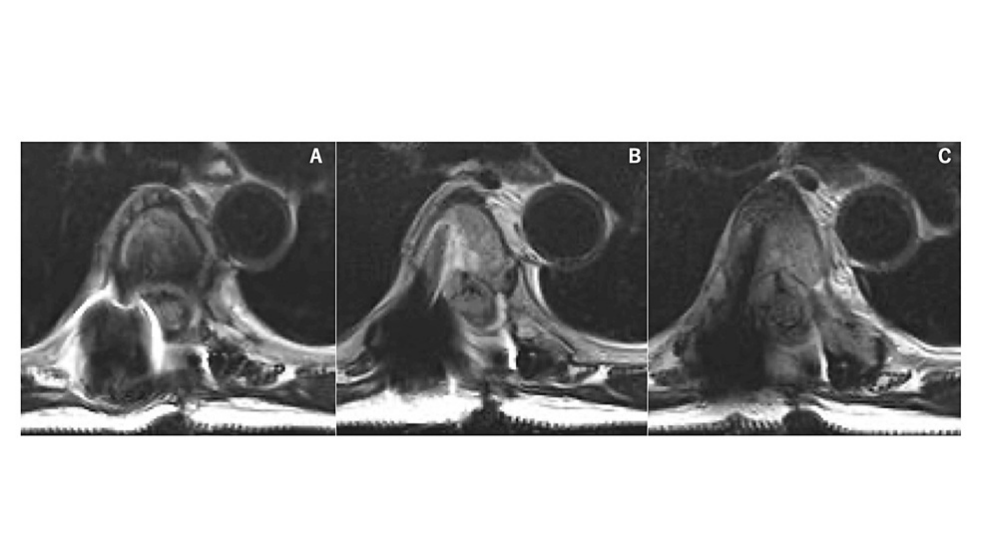
Magnetic resonance images after the third surgery After third tumor removal, spinal cord compression was improved, as seen in the MRI T2-weighted image at the Th5 level.

**Figure 6:**
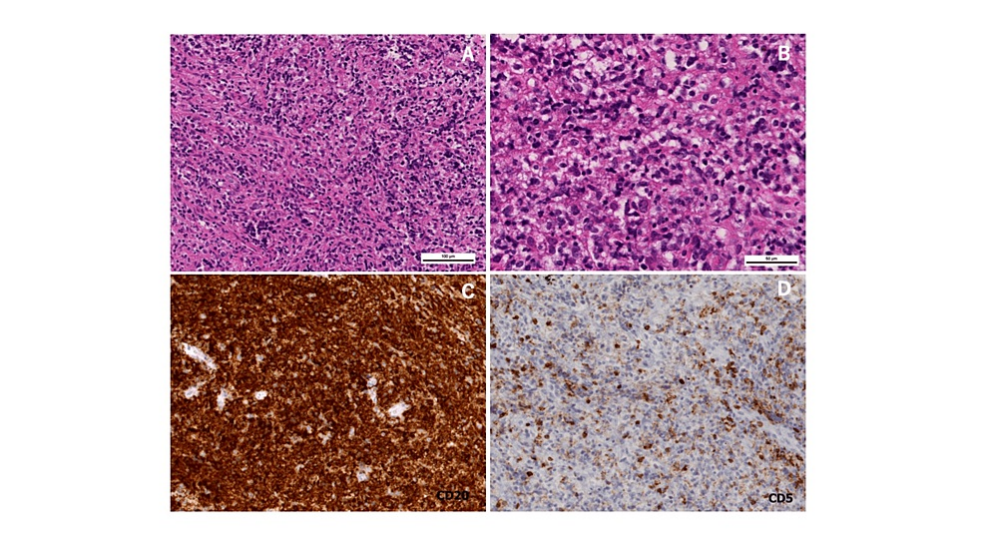
Second surgical pathological specimen The surgical specimen stained with hematoxylin-eosin shows atypical large lymphocytes compared to the previous specimen. (A: x100, B: x400). Immunohistochemical examinations revealed that the atypical cells were positive stained for CD20 (C) and negative stained for CD5 (D).

Three weeks later, the patient developed worsening paralysis of the lower limbs. A third surgical procedure was performed for tumor resection and spinal cord decompression combined with intraoperative ultrasound imaging. Histopathology of the third specimen conclusively identified diffuse large B-cell lymphoma (Figures 5, 6).

Unlike the initial pathology results, large atypical lymphocytes were observed. In immunostaining, CD20 was positive. Following this diagnosis, the patient was referred to a hematology team and chemotherapy was conducted. After two cycles of mini R-CHOP therapy, the patient exhibited significant functional improvement, regaining the ability to ambulate with the assistance of a walker, and was discharged to nursing facilities.

## Discussion

PBL is a rare disease, comprising merely 2% of all bone tumors and only 5% of all extra-nodal lymphomas [1,4]. Specifically, primary vertebral lesions account for only 1.7% of all PBL, predominantly observed in the thoracic or lumbar levels [5]. Tang et al. delineate the histological subtypes within PBL: diffuse large B cell lymphoma is 85%, T-cell lymphoma is 7%, plasmablastic lymphoma is 5%, NKT-cell lymphoma is 3% of the 40 patients studied [6]. 

Chisholm et al. highlighted the diagnostic challenges in PBL, noting that 30% of cases necessitated multiple biopsies due to issues such as necrosis, crush artifacts, or inadequate sample volume [1]. This high rate of repeat biopsies for PBL has previously been reported in other literature [7]. Furthermore, Tang et al. pointed out that needle biopsy is an important method for diagnosis, although the satisfactory positive rate of needle biopsy was hard to achieve. They reported the six patients who failed to achieve needle biopsy underwent surgical intervention [6]. In the presented case, PBL was definitively diagnosed in the third pathological examination following surgical intervention. This diagnosis stemmed from the observation of progressive lymphocyte atypia evident in the tissue samples obtained during this third evaluation. In our case, the first and second specimens included many necrosis parts, so diagnosis may have been difficult. In addition to this, large atypical lymphocytes increased gradually with progress, so we could diagnose PBL.

The optimal treatment of PBL of the spine is controversial. The treatments for PBL of the spine are chemotherapy, radiotherapy, and surgery. The main treatment is chemotherapy, but the rapid tumor progression often precipitates clinical complications. In such cases, surgical intervention is essential to ensure spinal stability [8]. Surgical resection of anterior spinal lesions is associated with great difficulty due to the complex anatomy and limitations in surgical access. To address these challenges, various advanced surgical support tools have been developed and documented in the literature. The utilization of CT-based navigation systems and O-arm navigation has been reported to be effective for these surgical resections [9,10]. In addition, intraoperative ultrasound has demonstrated considerable utility in delineating intradural conditions and anterior spinal cord lesions [11-13]. These integrated imaging approaches may allow for more accurate identification of tumor localization, potentially improving surgical outcomes and reducing the risk of unintentional spinal cord injury.

Conversely, Peng et al. reported that nonsurgical treatments with chemotherapy and radiotherapy rapidly reduced spinal cord and nerve root compression because PBL was a very chemo- and radio-sensitive tumor [3]. Nonetheless, Shibata et al. pointed out that spinal compression with vertebral fracture by tumor progression needs preventive spine reconstructions. If the paralysis is severe and progressive, early surgical decompression should be performed simultaneously with prophylactic spinal reconstruction, followed by chemical therapy [2].

## Conclusions

In the diagnostic evaluation of PBL of the spine, pathologic evaluation can often be challenging. Given the complexity of PBL, a singular biopsy often proves inadequate for a conclusive diagnosis. Integrated treatment approaches are necessary because PBL of the spine is difficult to address tumor progression with surgical intervention alone. Postoperative adjuvant therapy, especially chemotherapy and radiation therapy, is essential after a definitive diagnosis. Therefore, it is extremely important to obtain a definitive pathological diagnosis for this disease. If the diagnosis is difficult, we should consider multiple biopsies from different locations.
